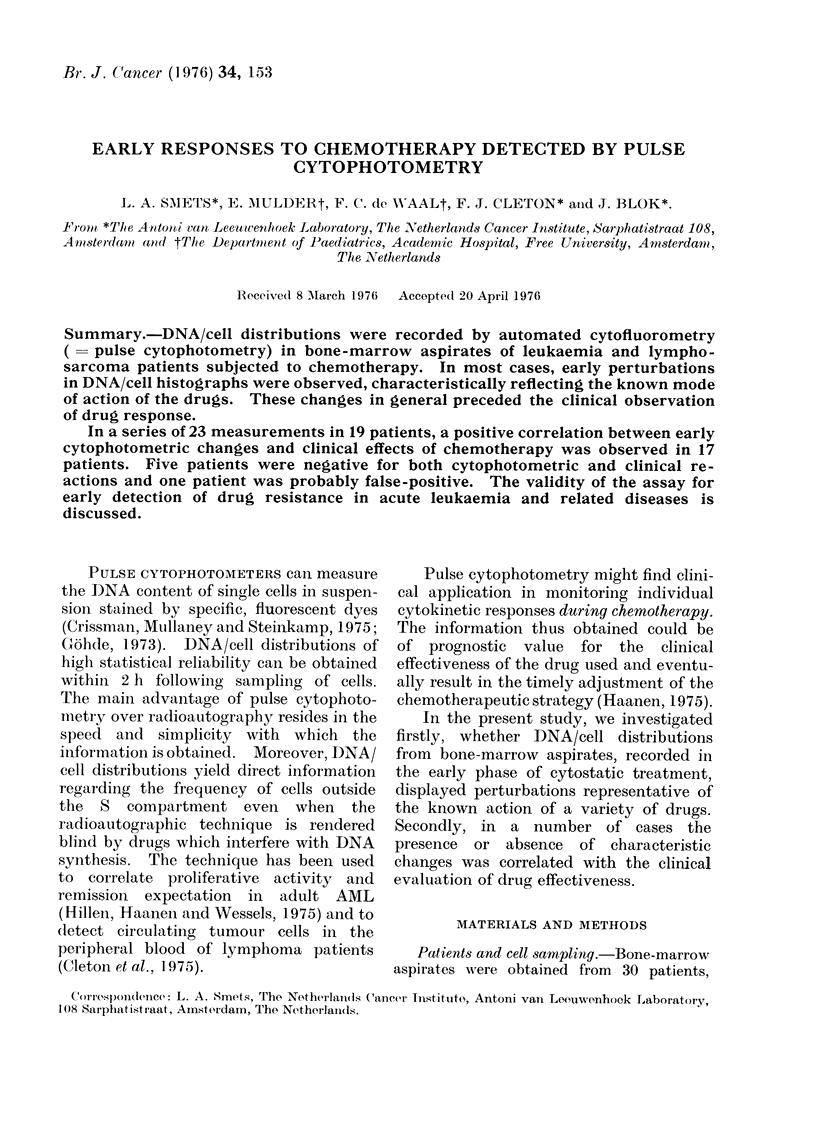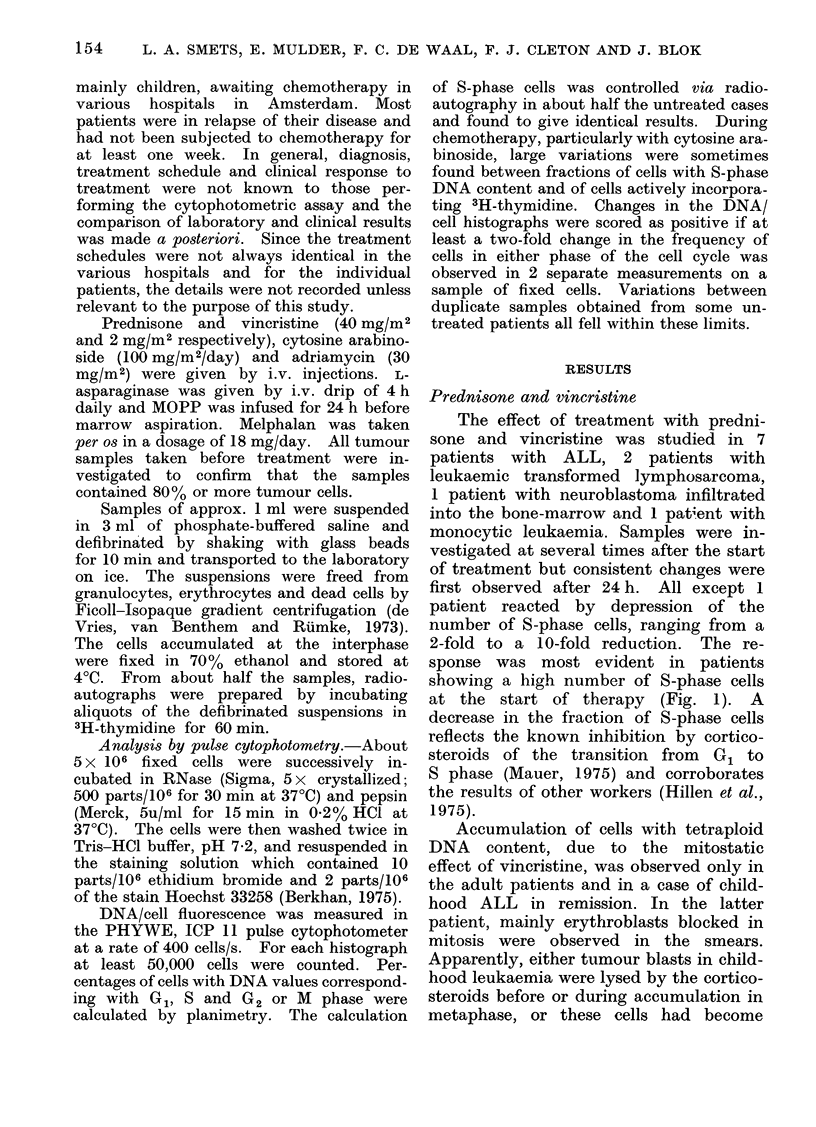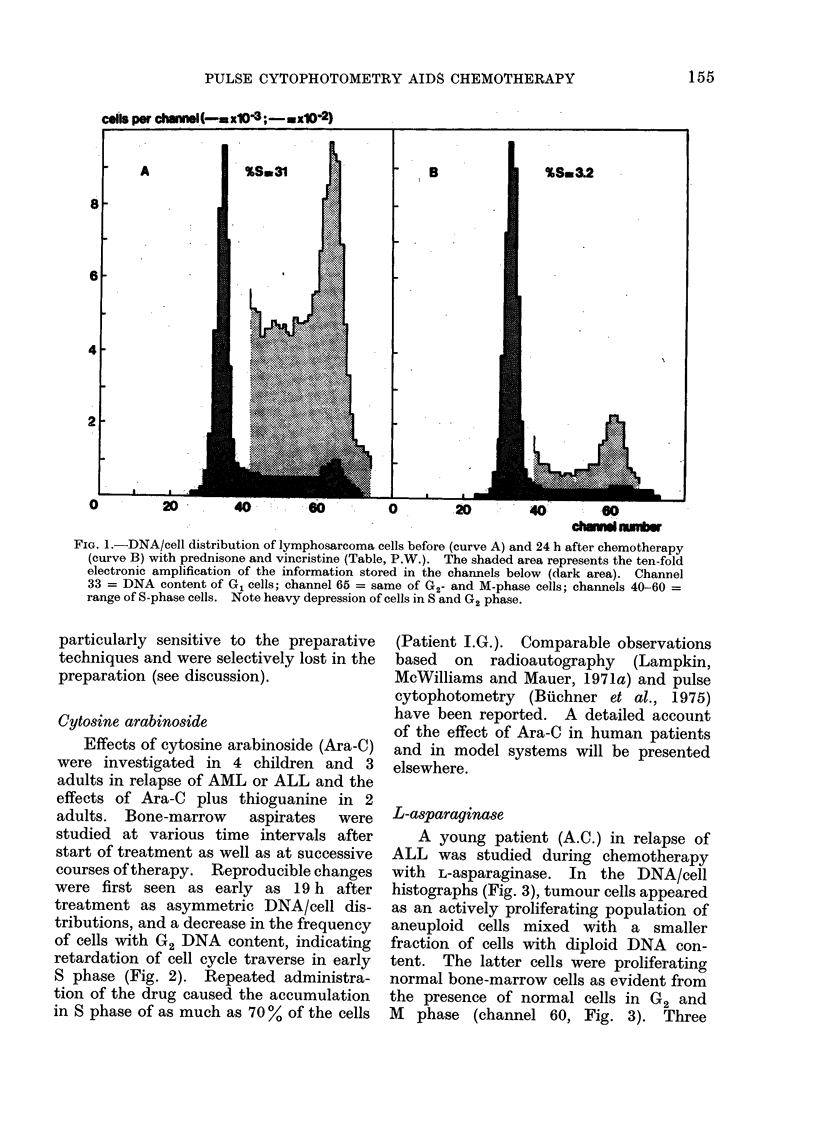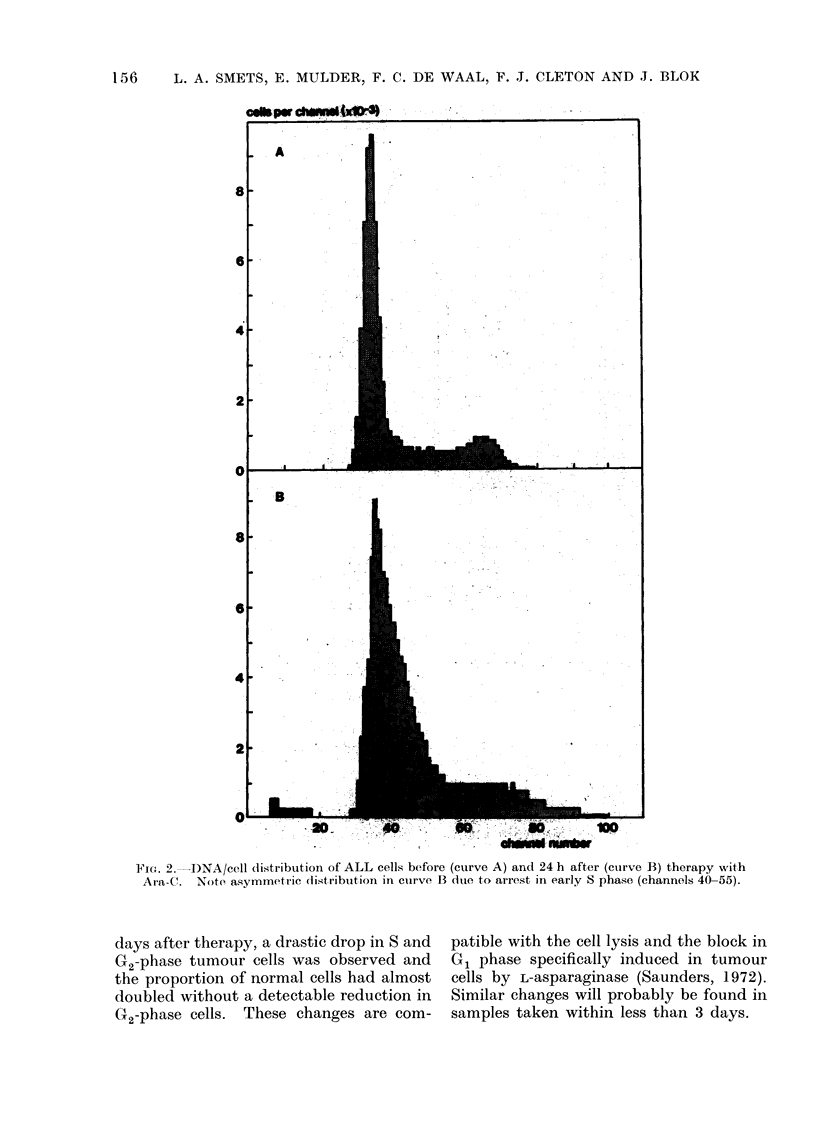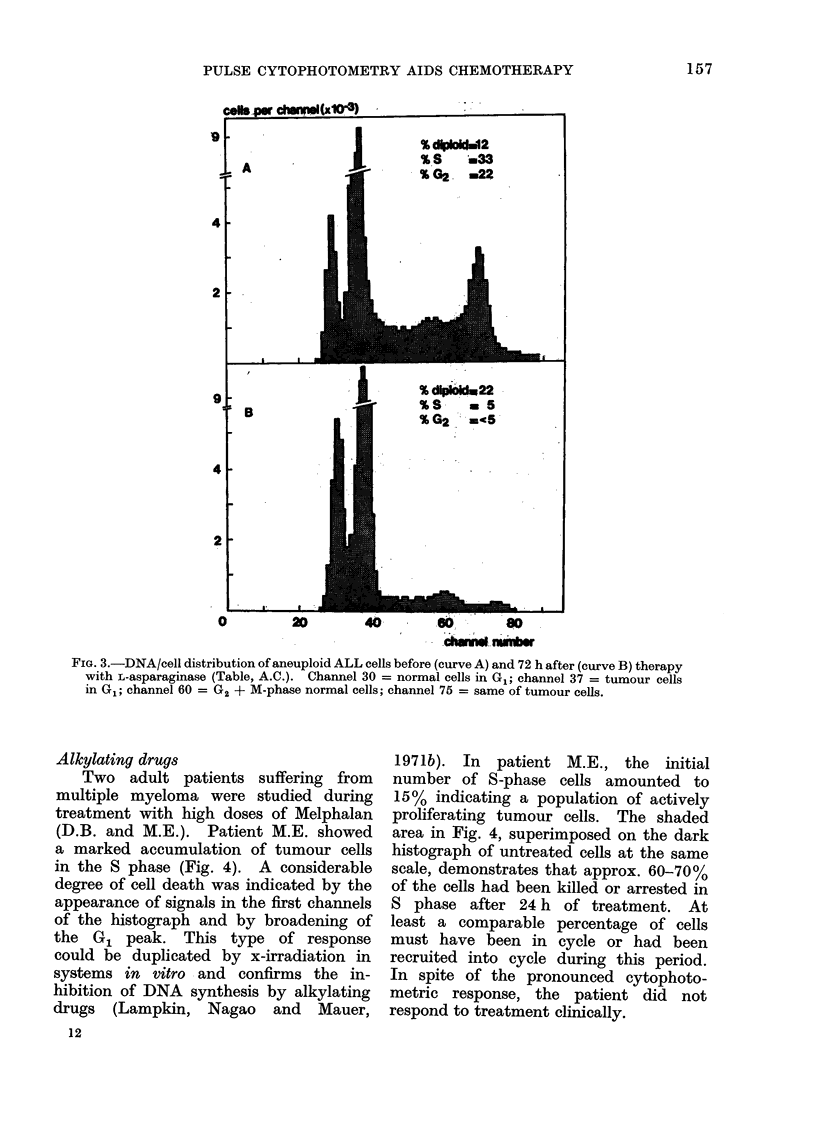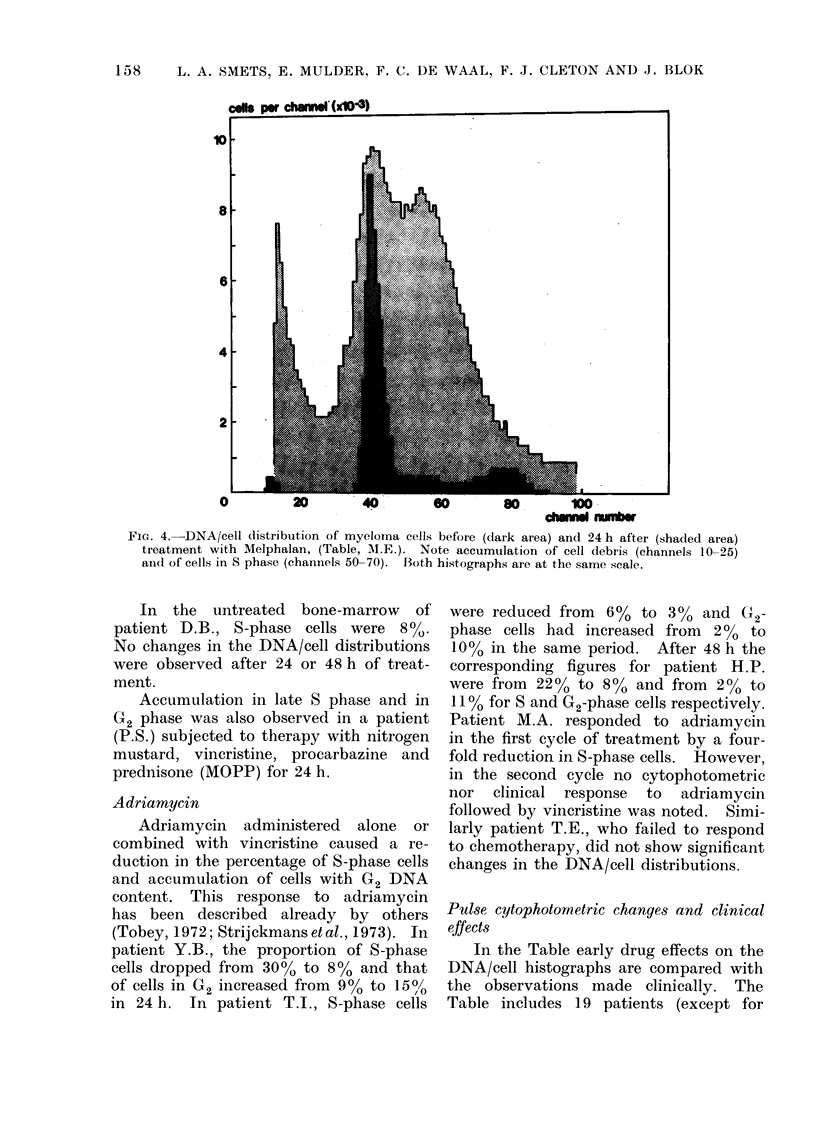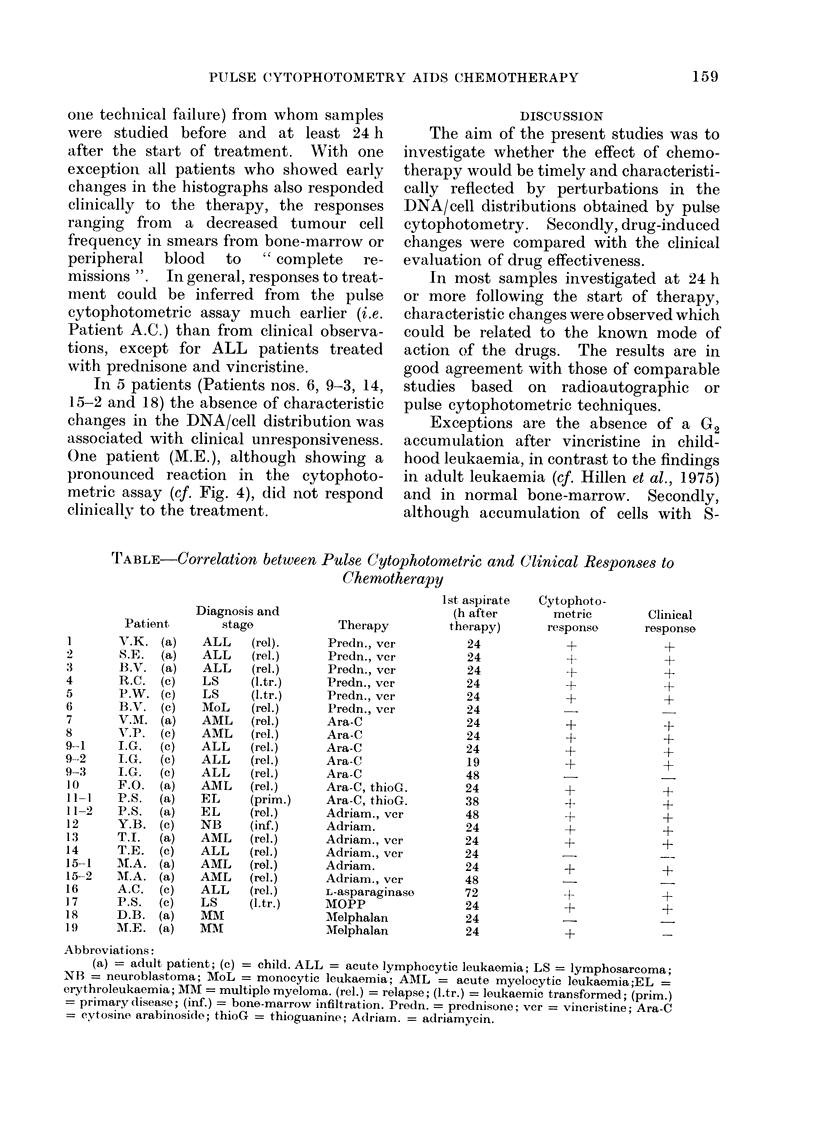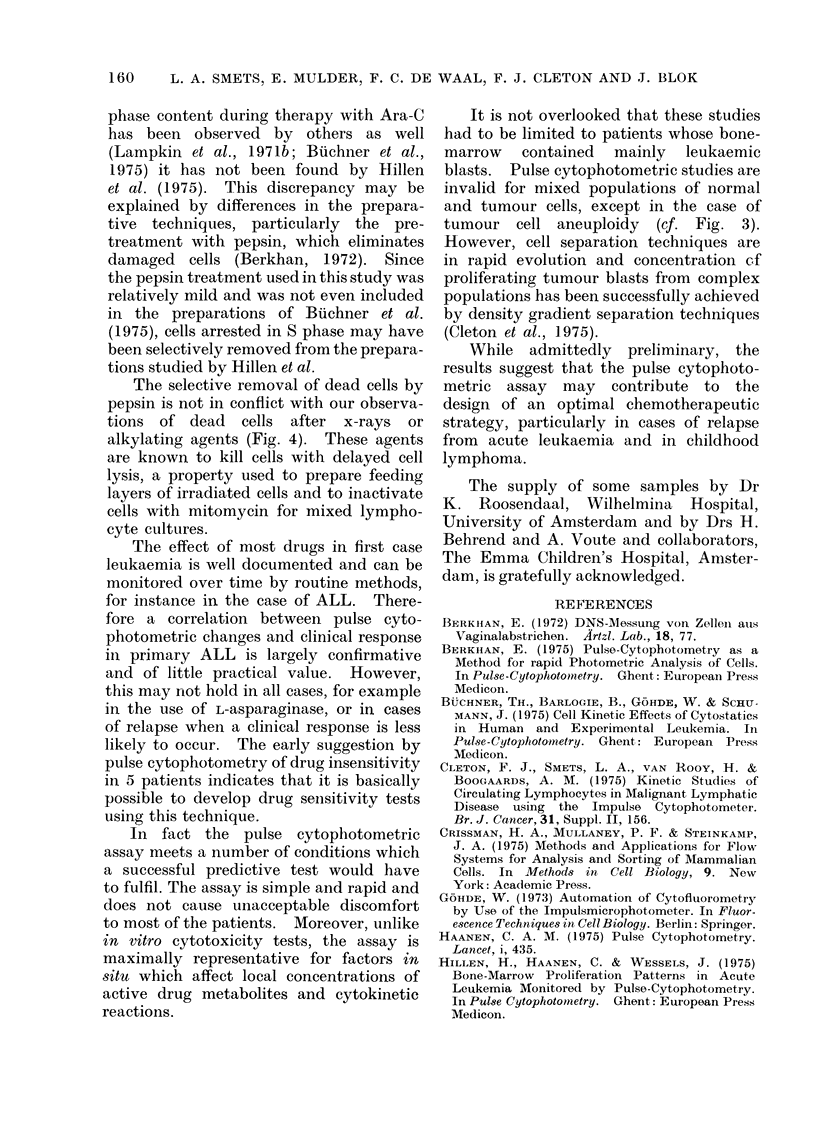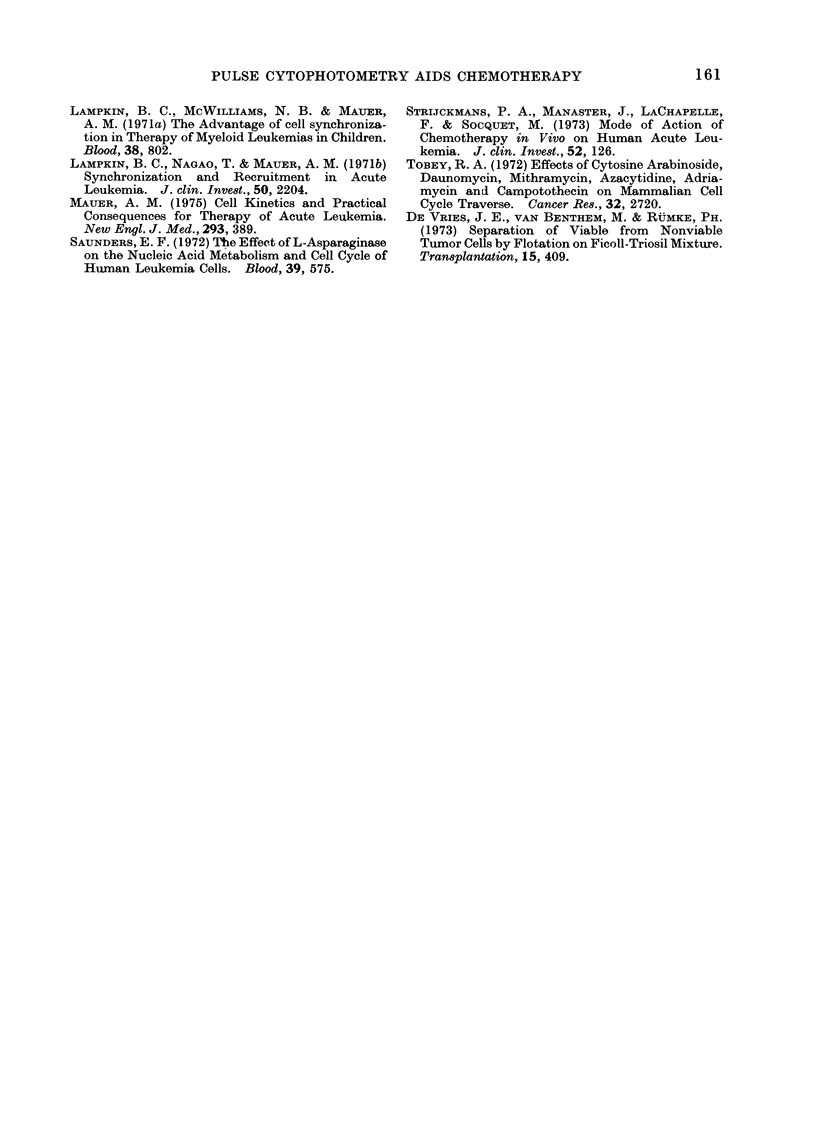# Early responses to chemotherapy detected by pulse cytophotometry.

**DOI:** 10.1038/bjc.1976.137

**Published:** 1976-08

**Authors:** L. A. Smets, E. Mulder, F. C. de Waal, F. J. Cleton, J. Blok

## Abstract

DNA/cell distributions were recorded by automated cytofluorometry (=pulse cytophotometry) in bone-marrow aspirates of leukaemia and lymphosarcoma patients subjected to chemotherapy. In most cases, early perturbations in DNA/cell histographs were observed, characteristically reflecting the known mode of action of the drugs. These changes in general preceded the clinical observation of drug response. In a series of 23 measurements in 19 patients, a positive correlation between early cytophotometric changes and clinical effects of chemotherapy was observed in 17 patients. Five patients were negative for both cytophotometric and clinical reactions and one patient was probably false-positive. The validity of the assay for early detection of drug resistance in acute leukaemia and related diseases is discussed.


*Br. J. Cancer* (1976) **34**, 153

### EARLY RESPONSES TO CHEMOTHERAPY DETECTED BY PULSE CYTOPHOTOMETRY

L. A. SMETS*, E. MULDER†, F. C. de WAAL†, F. J. CLETON* and J. BLOK*.

*From *The Antoni van Leeuwenhoek Laboratory, The Netherlands Cancer Institute, Sarphatistraat 108, Amsterdam and †The Department of Paediatrics, Academic Hospital, Free University, Amsterdam, The Netherlands*

Received 8 March 1976 Accepted 20 April 1976

**Summary.—DNA/cell distributions were recorded by automated cytofluorometry ( = pulse cytophotometry) in bone-marrow aspirates of leukaemia and lymphosarcoma patients subjected to chemotherapy. In most cases, early perturbations in DNA/cell histographs were observed, characteristically reflecting the known mode of action of the drugs. These changes in general preceded the clinical observation of drug response.**

**In a series of 23 measurements in 19 patients, a positive correlation between early cytophotometric changes and clinical effects of chemotherapy was observed in 17 patients. Five patients were negative for both cytophotometric and clinical reactions and one patient was probably false-positive. The validity of the assay for early detection of drug resistance in acute leukaemia and related diseases is discussed.**

PULSE CYTOPHOTOMETERS can measure the DNA content of single cells in suspension stained by specific, fluorescent dyes (Crissman, Mullaney and Steinkamp, 1975; Göhde, 1973). DNA/cell distributions of high statistical reliability can be obtained within 2 h following sampling of cells. The main advantage of pulse cytophotometry over radioautography resides in the speed and simplicity with which the information is obtained. Moreover, DNA/cell distributions yield direct information regarding the frequency of cells outside the S compartment even when the radioautographic technique is rendered blind by drugs which interfere with DNA synthesis. The technique has been used to correlate proliferative activity and remission expectation in adult AML (Hillen, Haanen and Wessels, 1975) and to detect circulating tumour cells in the peripheral blood of lymphoma patients (Cleton *et al.*, 1975).

Pulse cytophotometry might find clinical application in monitoring individual cytokinetic responses *during chemotherapy*. The information thus obtained could be of prognostic value for the clinical effectiveness of the drug used and eventually result in the timely adjustment of the chemotherapeutic strategy (Haanen, 1975).

In the present study, we investigated firstly, whether DNA/cell distributions from bone-marrow aspirates, recorded in the early phase of cytostatic treatment, displayed perturbations representative of the known action of a variety of drugs. Secondly, in a number of cases the presence or absence of characteristic changes was correlated with the clinical evaluation of drug effectiveness.

### MATERIALS AND METHODS

*Patients and cell sampling.*—Bone-marrow aspirates were obtained from 30 patients,

Correspondence: L. A. Smets, The Netherlands Cancer Institute, Antoni van Leeuwenhoek Laboratory, 108 Sarphatistraat, Amsterdam, The Netherlands.

mainly children, awaiting chemotherapy in various hospitals in Amsterdam. Most patients were in relapse of their disease and had not been subjected to chemotherapy for at least one week. In general, diagnosis, treatment schedule and clinical response to treatment were not known to those performing the cytophotometric assay and the comparison of laboratory and clinical results was made *a posteriori*. Since the treatment schedules were not always identical in the various hospitals and for the individual patients, the details were not recorded unless relevant to the purpose of this study.

Prednisone and vincristine (40 mg/m$^2$ and 2 mg/m$^2$ respectively), cytosine arabinoside (100 mg/m$^2$/day) and adriamycin (30 mg/m$^2$) were given by i.v. injections. L-asparaginase was given by i.v. drip of 4 h daily and MOPP was infused for 24 h before marrow aspiration. Melphalan was taken *per os* in a dosage of 18 mg/day. All tumour samples taken before treatment were investigated to confirm that the samples contained 80% or more tumour cells.

Samples of approx. 1 ml were suspended in 3 ml of phosphate-buffered saline and defibrinated by shaking with glass beads for 10 min and transported to the laboratory on ice. The suspensions were freed from granulocytes, erythrocytes and dead cells by Ficoll–Isopaque gradient centrifugation (de Vries, van Benthem and Rümke, 1973). The cells accumulated at the interphase were fixed in 70% ethanol and stored at 4°C. From about half the samples, radioautographs were prepared by incubating aliquots of the defibrinated suspensions in $^3$H-thymidine for 60 min.

*Analysis by pulse cytophotometry*.—About $5 \times 10^6$ fixed cells were successively incubated in RNase (Sigma, 5× crystallized; 500 parts/10$^6$ for 30 min at 37°C) and pepsin (Merck, 5u/ml for 15 min in 0·2% HCl at 37°C). The cells were then washed twice in Tris–HCl buffer, pH 7·2, and resuspended in the staining solution which contained 10 parts/10$^6$ ethidium bromide and 2 parts/10$^6$ of the stain Hoechst 33258 (Berkhan, 1975).

DNA/cell fluorescence was measured in the PHYWE, ICP 11 pulse cytophotometer at a rate of 400 cells/s. For each histograph at least 50,000 cells were counted. Percentages of cells with DNA values corresponding with $G_1$, S and $G_2$ or M phase were calculated by planimetry. The calculation of S-phase cells was controlled *via* radioautography in about half the untreated cases and found to give identical results. During chemotherapy, particularly with cytosine arabinoside, large variations were sometimes found between fractions of cells with S-phase DNA content and of cells actively incorporating $^3$H-thymidine. Changes in the DNA/cell histographs were scored as positive if at least a two-fold change in the frequency of cells in either phase of the cell cycle was observed in 2 separate measurements on a sample of fixed cells. Variations between duplicate samples obtained from some untreated patients all fell within these limits.

### RESULTS

### *Prednisone and vincristine*

The effect of treatment with prednisone and vincristine was studied in 7 patients with ALL, 2 patients with leukaemic transformed lymphosarcoma, 1 patient with neuroblastoma infiltrated into the bone-marrow and 1 patient with monocytic leukaemia. Samples were investigated at several times after the start of treatment but consistent changes were first observed after 24 h. All except 1 patient reacted by depression of the number of S-phase cells, ranging from a 2-fold to a 10-fold reduction. The response was most evident in patients showing a high number of S-phase cells at the start of therapy (Fig. 1). A decrease in the fraction of S-phase cells reflects the known inhibition by corticosteroids of the transition from $G_1$ to S phase (Mauer, 1975) and corroborates the results of other workers (Hillen *et al.*, 1975).

Accumulation of cells with tetraploid DNA content, due to the mitostatic effect of vincristine, was observed only in the adult patients and in a case of childhood ALL in remission. In the latter patient, mainly erythroblasts blocked in mitosis were observed in the smears. Apparently, either tumour blasts in childhood leukaemia were lysed by the corticosteroids before or during accumulation in metaphase, or these cells had become

FIG. 1.—DNA/cell distribution of lymphosarcoma cells before (curve A) and 24 h after chemotherapy (curve B) with prednisone and vincristine (Table, P.W.). The shaded area represents the ten-fold electronic amplification of the information stored in the channels below (dark area). Channel 33 = DNA content of $G_1$ cells; channel 65 = same of $G_2$- and M-phase cells; channels 40–60 = range of S-phase cells. Note heavy depression of cells in S and $G_2$ phase.

particularly sensitive to the preparative techniques and were selectively lost in the preparation (see discussion).

### *Cytosine arabinoside*

Effects of cytosine arabinoside (Ara-C) were investigated in 4 children and 3 adults in relapse of AML or ALL and the effects of Ara-C plus thioguanine in 2 adults. Bone-marrow aspirates were studied at various time intervals after start of treatment as well as at successive courses of therapy. Reproducible changes were first seen as early as 19 h after treatment as asymmetric DNA/cell distributions, and a decrease in the frequency of cells with $G_2$ DNA content, indicating retardation of cell cycle traverse in early S phase (Fig. 2). Repeated administration of the drug caused the accumulation in S phase of as much as 70% of the cells (Patient I.G.). Comparable observations based on radioautography (Lampkin, McWilliams and Mauer, 1971*a*) and pulse cytophotometry (Büchner *et al.*, 1975) have been reported. A detailed account of the effect of Ara-C in human patients and in model systems will be presented elsewhere.

### *L-asparaginase*

A young patient (A.C.) in relapse of ALL was studied during chemotherapy with L-asparaginase. In the DNA/cell histographs (Fig. 3), tumour cells appeared as an actively proliferating population of aneuploid cells mixed with a smaller fraction of cells with diploid DNA content. The latter cells were proliferating normal bone-marrow cells as evident from the presence of normal cells in $G_2$ and M phase (channel 60, Fig. 3). Three

FIG. 2.—DNA/cell distribution of ALL cells before (curve A) and 24 h after (curve B) therapy with Ara-C. Note asymmetric distribution in curve B due to arrest in early S phase (channels 40–55).

days after therapy, a drastic drop in S and $G_2$-phase tumour cells was observed and the proportion of normal cells had almost doubled without a detectable reduction in $G_2$-phase cells. These changes are compatible with the cell lysis and the block in $G_1$ phase specifically induced in tumour cells by L-asparaginase (Saunders, 1972). Similar changes will probably be found in samples taken within less than 3 days.

FIG. 3.—DNA/cell distribution of aneuploid ALL cells before (curve A) and 72 h after (curve B) therapy with L-asparaginase (Table, A.C.). Channel 30 = normal cells in $G_1$; channel 37 = tumour cells in $G_1$; channel 60 = $G_2$ + M-phase normal cells; channel 75 = same of tumour cells.

*Alkylating drugs*

Two adult patients suffering from multiple myeloma were studied during treatment with high doses of Melphalan (D.B. and M.E.). Patient M.E. showed a marked accumulation of tumour cells in the S phase (Fig. 4). A considerable degree of cell death was indicated by the appearance of signals in the first channels of the histograph and by broadening of the $G_1$ peak. This type of response could be duplicated by x-irradiation in systems *in vitro* and confirms the inhibition of DNA synthesis by alkylating drugs (Lampkin, Nagao and Mauer, 1971*b*). In patient M.E., the initial number of S-phase cells amounted to 15% indicating a population of actively proliferating tumour cells. The shaded area in Fig. 4, superimposed on the dark histograph of untreated cells at the same scale, demonstrates that approx. 60–70% of the cells had been killed or arrested in S phase after 24 h of treatment. At least a comparable percentage of cells must have been in cycle or had been recruited into cycle during this period. In spite of the pronounced cytophotometric response, the patient did not respond to treatment clinically.

FIG. 4.—DNA/cell distribution of myeloma cells before (dark area) and 24 h after (shaded area) treatment with Melphalan, (Table, M.E.). Note accumulation of cell debris (channels 10–25) and of cells in S phase (channels 50–70). Both histographs are at the same scale.

In the untreated bone-marrow of patient D.B., S-phase cells were 8%. No changes in the DNA/cell distributions were observed after 24 or 48 h of treatment.

Accumulation in late S phase and in $G_2$ phase was also observed in a patient (P.S.) subjected to therapy with nitrogen mustard, vincristine, procarbazine and prednisone (MOPP) for 24 h.

*Adriamycin*

Adriamycin administered alone or combined with vincristine caused a reduction in the percentage of S-phase cells and accumulation of cells with $G_2$ DNA content. This response to adriamycin has been described already by others (Tobey, 1972; Strijckmans *et al.*, 1973). In patient Y.B., the proportion of S-phase cells dropped from 30% to 8% and that of cells in $G_2$ increased from 9% to 15% in 24 h. In patient T.I., S-phase cells were reduced from 6% to 3% and $G_2$-phase cells had increased from 2% to 10% in the same period. After 48 h the corresponding figures for patient H.P. were from 22% to 8% and from 2% to 11% for S and $G_2$-phase cells respectively. Patient M.A. responded to adriamycin in the first cycle of treatment by a fourfold reduction in S-phase cells. However, in the second cycle no cytophotometric nor clinical response to adriamycin followed by vincristine was noted. Similarly patient T.E., who failed to respond to chemotherapy, did not show significant changes in the DNA/cell distributions.

*Pulse cytophotometric changes and clinical effects*

In the Table early drug effects on the DNA/cell histographs are compared with the observations made clinically. The Table includes 19 patients (except for one technical failure) from whom samples were studied before and at least 24 h after the start of treatment. With one exception all patients who showed early changes in the histographs also responded clinically to the therapy, the responses ranging from a decreased tumour cell frequency in smears from bone-marrow or peripheral blood to " complete remissions ". In general, responses to treatment could be inferred from the pulse cytophotometric assay much earlier (*i.e.* Patient A.C.) than from clinical observations, except for ALL patients treated with prednisone and vincristine.

In 5 patients (Patients nos. 6, 9–3, 14, 15–2 and 18) the absence of characteristic changes in the DNA/cell distribution was associated with clinical unresponsiveness. One patient (M.E.), although showing a pronounced reaction in the cytophotometric assay (*cf.* Fig. 4), did not respond clinically to the treatment.

### DISCUSSION

The aim of the present studies was to investigate whether the effect of chemotherapy would be timely and characteristically reflected by perturbations in the DNA/cell distributions obtained by pulse cytophotometry. Secondly, drug-induced changes were compared with the clinical evaluation of drug effectiveness.

In most samples investigated at 24 h or more following the start of therapy, characteristic changes were observed which could be related to the known mode of action of the drugs. The results are in good agreement with those of comparable studies based on radioautographic or pulse cytophotometric techniques.

Exceptions are the absence of a $G_2$ accumulation after vincristine in childhood leukaemia, in contrast to the findings in adult leukaemia (*cf.* Hillen *et al.*, 1975) and in normal bone-marrow. Secondly, although accumulation of cells with S-

TABLE—*Correlation between Pulse Cytophotometric and Clinical Responses to Chemotherapy*

| | Patient | | Diagnosis and stage | | Therapy | 1st aspirate (h after therapy) | Cytophotometric response | Clinical response |
|---|---|---|---|---|---|---|---|---|
| 1 | V.K. | (a) | ALL | (rel). | Predn., vcr | 24 | + | + |
| 2 | S.E. | (a) | ALL | (rel.) | Predn., vcr | 24 | + | + |
| 3 | B.V. | (a) | ALL | (rel.) | Predn., vcr | 24 | + | + |
| 4 | R.C. | (c) | LS | (l.tr.) | Predn., vcr | 24 | + | + |
| 5 | P.W. | (c) | LS | (l.tr.) | Predn., vcr | 24 | + | + |
| 6 | B.V. | (c) | MoL | (rel.) | Predn., vcr | 24 | — | — |
| 7 | V.M. | (a) | AML | (rel.) | Ara-C | 24 | + | + |
| 8 | V.P. | (c) | AML | (rel.) | Ara-C | 24 | + | + |
| 9–1 | I.G. | (c) | ALL | (rel.) | Ara-C | 24 | + | + |
| 9–2 | I.G. | (c) | ALL | (rel.) | Ara-C | 19 | + | + |
| 9–3 | I.G. | (c) | ALL | (rel.) | Ara-C | 48 | — | — |
| 10 | F.O. | (a) | AML | (rel.) | Ara-C, thioG. | 24 | + | + |
| 11–1 | P.S. | (a) | EL | (prim.) | Ara-C, thioG. | 38 | + | + |
| 11–2 | P.S. | (a) | EL | (rel.) | Adriam., vcr | 48 | + | + |
| 12 | Y.B. | (c) | NB | (inf.) | Adriam. | 24 | + | + |
| 13 | T.I. | (a) | AML | (rel.) | Adriam., vcr | 24 | + | + |
| 14 | T.E. | (c) | ALL | (rel.) | Adriam., vcr | 24 | — | — |
| 15–1 | M.A. | (a) | AML | (rel.) | Adriam. | 24 | + | + |
| 15–2 | M.A. | (a) | AML | (rel.) | Adriam., vcr | 48 | — | — |
| 16 | A.C. | (c) | ALL | (rel.) | L-asparaginase | 72 | + | + |
| 17 | P.S. | (c) | LS | (l.tr.) | MOPP | 24 | + | + |
| 18 | D.B. | (a) | MM | | Melphalan | 24 | — | — |
| 19 | M.E. | (a) | MM | | Melphalan | 24 | + | — |

Abbreviations:
(a) = adult patient; (c) = child. ALL = acute lymphocytic leukaemia; LS = lymphosarcoma; NB = neuroblastoma; MoL = monocytic leukaemia; AML = acute myelocytic leukaemia;EL = erythroleukaemia; MM = multiple myeloma. (rel.) = relapse; (l.tr.) = leukaemic transformed; (prim.) = primary disease; (inf.) = bone-marrow infiltration. Predn. = prednisone; vcr = vincristine; Ara-C = cytosine arabinoside; thioG = thioguanine; Adriam. = adriamycin.

phase content during therapy with Ara-C has been observed by others as well (Lampkin *et al.*, 1971*b*; Büchner *et al.*, 1975) it has not been found by Hillen *et al.* (1975). This discrepancy may be explained by differences in the preparative techniques, particularly the pretreatment with pepsin, which eliminates damaged cells (Berkhan, 1972). Since the pepsin treatment used in this study was relatively mild and was not even included in the preparations of Büchner *et al.* (1975), cells arrested in S phase may have been selectively removed from the preparations studied by Hillen *et al.*

The selective removal of dead cells by pepsin is not in conflict with our observations of dead cells after x-rays or alkylating agents (Fig. 4). These agents are known to kill cells with delayed cell lysis, a property used to prepare feeding layers of irradiated cells and to inactivate cells with mitomycin for mixed lymphocyte cultures.

The effect of most drugs in first case leukaemia is well documented and can be monitored over time by routine methods, for instance in the case of ALL. Therefore a correlation between pulse cytophotometric changes and clinical response in primary ALL is largely confirmative and of little practical value. However, this may not hold in all cases, for example in the use of L-asparaginase, or in cases of relapse when a clinical response is less likely to occur. The early suggestion by pulse cytophotometry of drug insensitivity in 5 patients indicates that it is basically possible to develop drug sensitivity tests using this technique.

In fact the pulse cytophotometric assay meets a number of conditions which a successful predictive test would have to fulfil. The assay is simple and rapid and does not cause unacceptable discomfort to most of the patients. Moreover, unlike *in vitro* cytotoxicity tests, the assay is maximally representative for factors *in situ* which affect local concentrations of active drug metabolites and cytokinetic reactions.

It is not overlooked that these studies had to be limited to patients whose bone-marrow contained mainly leukaemic blasts. Pulse cytophotometric studies are invalid for mixed populations of normal and tumour cells, except in the case of tumour cell aneuploidy (*cf.* Fig. 3). However, cell separation techniques are in rapid evolution and concentration of proliferating tumour blasts from complex populations has been successfully achieved by density gradient separation techniques (Cleton *et al.*, 1975).

While admittedly preliminary, the results suggest that the pulse cytophotometric assay may contribute to the design of an optimal chemotherapeutic strategy, particularly in cases of relapse from acute leukaemia and in childhood lymphoma.

The supply of some samples by Dr K. Roosendaal, Wilhelmina Hospital, University of Amsterdam and by Drs H. Behrend and A. Voute and collaborators, The Emma Children's Hospital, Amsterdam, is gratefully acknowledged.

### REFERENCES

BERKHAN, E. (1972) DNS-Messung von Zellen aus Vaginalabstrichen. *Ärtzl. Lab.*, **18**, 77.

BERKHAN, E. (1975) Pulse-Cytophotometry as a Method for rapid Photometric Analysis of Cells. In *Pulse-Cytophotometry*. Ghent: European Press Medicon.

BÜCHNER, TH., BARLOGIE, B., GÖHDE, W. & SCHUMANN, J. (1975) Cell Kinetic Effects of Cytostatics in Human and Experimental Leukemia. In *Pulse-Cytophotometry*. Ghent: European Press Medicon.

CLETON, F. J., SMETS, L. A., VAN ROOY, H. & BOOGAARDS, A. M. (1975) Kinetic Studies of Circulating Lymphocytes in Malignant Lymphatic Disease using the Impulse Cytophotometer. *Br. J. Cancer*, **31**, Suppl. II, 156.

CRISSMAN, H. A., MULLANEY, P. F. & STEINKAMP, J. A. (1975) Methods and Applications for Flow Systems for Analysis and Sorting of Mammalian Cells. In *Methods in Cell Biology*, **9**. New York: Academic Press.

GÖHDE, W. (1973) Automation of Cytofluorometry by Use of the Impulsmicrophotometer. In *Fluorescence Techniques in Cell Biology*. Berlin: Springer.

HAANEN, C. A. M. (1975) Pulse Cytophotometry. *Lancet*, i, 435.

HILLEN, H., HAANEN, C. & WESSELS, J. (1975) Bone-Marrow Proliferation Patterns in Acute Leukemia Monitored by Pulse-Cytophotometry. In *Pulse Cytophotometry*. Ghent: European Press Medicon.

LAMPKIN, B. C., MCWILLIAMS, N. B. & MAUER, A. M. (1971*a*) The Advantage of cell synchronization in Therapy of Myeloid Leukemias in Children. *Blood*, **38**, 802.

LAMPKIN, B. C., NAGAO, T. & MAUER, A. M. (1971*b*) Synchronization and Recruitment in Acute Leukemia. *J. clin. Invest.*, **50**, 2204.

MAUER, A. M. (1975) Cell Kinetics and Practical Consequences for Therapy of Acute Leukemia. *New Engl. J. Med.*, **293**, 389.

SAUNDERS, E. F. (1972) The Effect of L-Asparaginase on the Nucleic Acid Metabolism and Cell Cycle of Human Leukemia Cells. *Blood*, **39**, 575.

STRIJCKMANS, P. A., MANASTER, J., LACHAPELLE, F. & SOCQUET, M. (1973) Mode of Action of Chemotherapy *in Vivo* on Human Acute Leukemia. *J. clin. Invest.*, **52**, 126.

TOBEY, R. A. (1972) Effects of Cytosine Arabinoside, Daunomycin, Mithramycin, Azacytidine, Adriamycin and Campotothecin on Mammalian Cell Cycle Traverse. *Cancer Res.*, **32**, 2720.

DE VRIES, J. E., VAN BENTHEM, M. & RÜMKE, PH. (1973) Separation of Viable from Nonviable Tumor Cells by Flotation on Ficoll-Triosil Mixture. *Transplantation*, **15**, 409.